# The Potential for Transition Metal-Mediated Neurodegeneration in Amyotrophic Lateral Sclerosis

**DOI:** 10.3389/fnagi.2014.00173

**Published:** 2014-07-23

**Authors:** David B. Lovejoy, Gilles J. Guillemin

**Affiliations:** ^1^Australian School of Advanced Medicine, Macquarie University, Sydney, NSW, Australia

**Keywords:** amyotrophic lateral sclerosis, neurodegeneration, transition metals, iron, copper, ER stress

## Abstract

Modulations of the potentially toxic transition metals iron (Fe) and copper (Cu) are implicated in the neurodegenerative process in a variety of human disease states including amyotrophic lateral sclerosis (ALS). However, the precise role played by these metals is still very much unclear, despite considerable clinical and experimental data suggestive of a role for these elements in the neurodegenerative process. The discovery of mutations in the antioxidant enzyme Cu/Zn superoxide dismutase 1 (SOD-1) in ALS patients established the first known cause of ALS. Recent data suggest that various mutations in SOD-1 affect metal-binding of Cu and Zn, in turn promoting toxic protein aggregation. Copper homeostasis is also disturbed in ALS, and may be relevant to ALS pathogenesis. Another set of interesting observations in ALS patients involves the key nutrient Fe. In ALS patients, Fe loading can be inferred by studies showing increased expression of serum ferritin, an Fe-storage protein, with high serum ferritin levels correlating to poor prognosis. Magnetic resonance imaging of ALS patients shows a characteristic T_2_ shortening that is attributed to the presence of Fe in the motor cortex. In mutant SOD-1 mouse models, increased Fe is also detected in the spinal cord and treatment with Fe-chelating drugs lowers spinal cord Fe, preserves motor neurons, and extends lifespan. Inflammation may play a key causative role in Fe accumulation, but this is not yet conclusive. Excess transition metals may enhance induction of endoplasmic reticulum (ER) stress, a system that is already under strain in ALS. Taken together, the evidence suggests a role for transition metals in ALS progression and the potential use of metal-chelating drugs as a component of future ALS therapy.

## Introduction

The burden of amyotrophic lateral sclerosis (ALS) is very significant with a world-wide disease incidence of 2 in 100,000 (Chio et al., 2013). It is a rapidly debilitating neuromuscular disorder characterized by loss of motor neurons from the cerebral cortex, brainstem, and spinal cord. Progressive muscle weakness, wasting and paralysis then follow with death typically occurring 3–5 years post-diagnosis. Approximately 95–90% of ALS cases are sporadic (sALS) while the balance is genetically linked familial disease (fALS), where mutations in superoxide dismutase 1 (SOD-1) cause 25% of cases. At least 160 mutations in SOD-1 have been associated with fALS and sALS (Sreedharan and Brown, 2013).

With the possible exception of mutant SOD-1, which forms toxic protein aggregates, the etiology of this disease is still unclear. Other postulated mechanisms of neuronal death in ALS include glutamate excitotoxicity (Rothstein, 2009), excessive generation of reactive oxygen and nitrogen species (Barber and Shaw, 2010), mitochondrial dysfunction (Rothstein, 2009), induction of endoplasmic reticulum (ER) stress (Atkin et al., 2008), axonal deterioration, and deposition of toxic ubiquitinated neuronal inclusions, where transactive response DNA binding protein 43 kDa (TDP-43) and fused in sarcoma (FUS) are major protein components (Arai et al., 2006; Rothstein, 2009). Many of these mechanisms are inter-connected and “snowballing” effects can be envisaged where events trigger, and/or exacerbate, others.

A common thread that links a variety of neurodegenerative conditions is the build-up of the transition metals iron (Fe) and copper (Cu) in the CNS (for review see Hadzhieva et al., 2013a; Jellinger, 2013). Ordinarily, these metals are incorporated into a wide range of vital enzymes, as their redox capability enables an efficient biochemical “switch” function, but this means that their homeostasis must be tightly regulated in order to prevent excessive production of reactive oxygen species that can damage cellular components. Alzheimer’s diseases (AD) and Parkinson’s diseases (PD), in particular, show marked brain and/or CNS increases in these metals and much attention has focused on the role of these metals in these conditions.

In AD, Fe and Cu are thought to play roles in the formation of amyloid-β plaques and tau neurofibrillary tangles (Hane et al., 2013; Savelieff et al., 2013) and recent magnetic resonance image (MRI) studies showed elevated Fe in the hippocampus in AD patients correlating to decreased structural integrity of the hippocampus, possibly caused by a process of demyelination (Raven et al., 2013). An interesting speculation by these authors was that demyelination could generate a vicious cycle of further Fe deposition as myelin contains significant Fe. However, there is still controversy surrounding the role of Fe in AD, as others have failed to find elevated Fe in AD brain, leading to the suggestion that a citation bias exists in the literature favoring studies reporting increased brain Fe in AD (Schrag et al., 2011).

Accumulation of α-synuclein in Lewy bodies in the substantia nigra is the primary pathological hallmark of PD and results in the death of dopaminergic neurons. A variety of experimental evidences suggest that Fe and Cu not only facilitate aggregation of α-synuclein, but also potentiate the toxicity of these deposits (Santner and Uversky, 2010; Rose et al., 2011).

Despite controversy, chelation therapy targeting Fe and/or Cu has been advocated for AD and PD with encouraging results in some AD clinical trials with the chelators desferrioxamine (Crapper McLachlan et al., 1991) and clioquinol (Ritchie et al., 2003). However, the clinical value of lowering metals in these conditions still requires further validation.

A variety of other neurodegenerative disorders also show disturbances in Fe and/or Cu metabolism, leading to excess (or reduced) loading of these metals. These include the other α-synucleinopathies, a variety of disorders grouped together as “neurodegeneration with brain iron accumulation” (NBIA), multiple sclerosis, Huntington’s disease, Wilson’s disease, Menkes disease, Friedreich’s ataxia, and the prion diseases (for reviews see Spillantini and Goedert, 2000; Hagemeier et al., 2012; Rouault, 2013).

That Fe, Cu, and zinc (Zn) may have a role in the pathophysiology of human ALS is highlighted by a diverse range of recent studies (2011–2013) showing that (a) higher concentrations of serum ferritin (an Fe-storage protein reflecting high body Fe levels) correlate to poor prognosis in ALS patients (Ikeda et al., 2012; Nadjar et al., 2012); (b) Fe accumulation in the motor cortex is responsible for frequently observed MRI abnormalities in ALS patients (Langkammer et al., 2010; Kwan et al., 2012; Ignjatovic et al., 2013); (c) inappropriately chelated Fe (i.e., capable of promoting free-radical generation) was found in the CSF of ALS patients but not normal controls (Ignjatovic et al., 2012); (d) in a group of 51 ALS patients, the levels of magnesium (Mg) (*p* < 0.01), Fe (*p* < 0.05), Cu (*p* < 0.05), and Zn (*p* < 0.10) in CSF were higher than those in controls, with some patients showing very high levels of Cu and Zn before showing critical clinical deterioration (Hozumi et al., 2011). These studies build on a multitude of others that have shown a variety of disturbances in Fe/Cu/Zn metabolism leading to metal deposition or dysfunction of key metalloenzymes in ALS (Oshiro et al., 2011).

Also of interest is the link between mutations in the hemochromatosis gene (HFE; principally the H63D polymorphism) and sALS; mutations which are thought to induce prolonged ER stress possibly through an Fe-overloading mechanism (Liu et al., 2011b). Other observations also support the possibility that excess redox-active metals may enhance ER stress in ALS by perturbing redox status, disrupting normal protein folding, and modulating ER/cytosolic calcium balance.

ALS and other neurodegenerative conditions are also accompanied by chronic neuroinflammation. Evidence shows that inflammation modulates the expression of several key proteins of Fe homeostasis, and can result in further neuronal Fe accumulation, but this potentially significant inter-play has yet to be fully explored in ALS.

Providing a therapeutic proof-of-principle argument in the role of these metals in ALS are studies in SOD-1 mutant mice where at least three different Fe (Jeong et al., 2009; Wang et al., 2011) and five Cu/Zn-chelating agents have shown significant rescue effects (Hottinger et al., 1997; Nagano et al., 2003; Petri et al., 2007; Tokuda et al., 2008). Several chelators were also able to reverse, in part, the dysregulation of Fe or Cu in SOD-1 models, reducing microglial activation that seems to occasion neuronal loss and significantly extended lifespan. Overexpression of the endogenous metal-binding protein, metallothionein (MT) (Tokuda et al., 2014), or treatment with the Cu-chelate and Cu(II)-ASTM (Soon et al., 2011) also increased survival of mutant SOD-1 mice, demonstrating a variety of rescue effects.

Taken together, these divergent data suggest that transition metals play some role in the neurodegenerative process in ALS. However, considerable gaps in our knowledge remain and in this review we seek to provide an overview of the latest research together with perspectives on future research and clinical implications.

## Fe Metabolism in the Brain and CNS: General Overview

**Figure 1:**
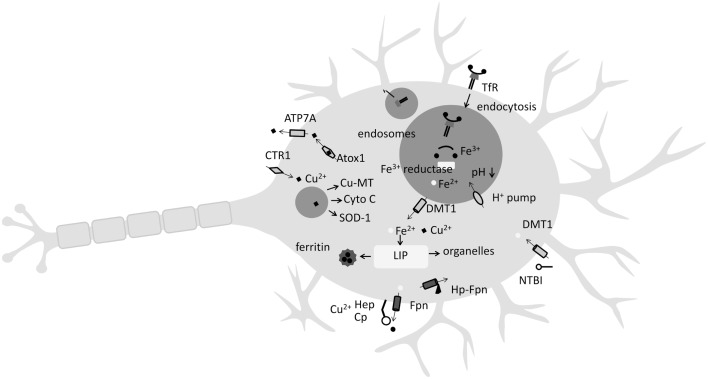
**Key features of Fe and Cu uptake and release by the neuron**. Iron (Fe) is either acquired by non-transferrin bound Fe (NTBI) from low-molecular weight complexes with citrate or ascorbate or by endocytosis of the transferrin (Tf)-transferrin receptor complex (TfR). Once endocytosed, a decrease in pH and the action of a ferrioxidase enables Fe^2+^ release to the labile Fe pool (LIP) by divalent metal transporter 1 (DMT1) where it may be stored in the Fe-storage protein ferritin, or directed to organelles such as lysosomes. Copper (Cu) uptake occurs at neuron surface by copper transporter-1 (CTR1). Following endocytosis, Cu^2+^ ions are incorporated into Cu-metallothionein (Cu-MT), cytochrome *c*, or superoxide dismutase 1 (SOD-1). Ferroportin (Fpn), the only known Fe exporter from neurons, relies on the Cu-containing metalloenzymes hephaestin (Hep) or ceruloplasmin (Cp) for activity. Hepcidin (Hp) activity can internalize Fpn.

Iron homeostasis in the body begins by tight control of Fe release from enterocytes in the small intestine by the hormone hepcidin (Hp). Generally acknowledged as the master regulator of Fe homeostasis, Hp prevents enterocyte Fe release into the blood by binding to ferroportin-1 (Fpn). It is produced mainly by hepatocytes but is also expressed in mouse and human CNS and brain (Zechel et al., 2006; Wang et al., 2010). The major blood Fe-transport molecule is transferrin (Tf) and most cell types, including neurons, acquire Fe by a process where plasma Tf complexes with Tf-receptors (TfR) on the cell surface with subsequent internalization by endocytosis (Figure 1). Following Tf–TfR complex internalization to endosomes, where lower pH results in the release of Fe^3+^ from Tf, a ferric reductase, possibly six-transmembrane epithelial antigen of the prostate family member 3 (STEAP3), or duodenal cytochrome B (dcytb) then reduces Fe^3+^ to Fe^2+^ facilitating binding to divalent metal transporter 1 (DMT1) and endosomal export to the cytosol (Figure 1). In terms of the brain, plasma Tf and other proteins of Fe metabolism do not cross the blood–brain barrier (BBB). Hence, an additional CNS Fe-acquisition step is required, essentially an iteration of the above process, where plasma Tf binds to TfR present on the luminal membrane of cerebrovascular endothelial cells. Following cytosolic release, Fe is exported across the endothelial membrane into interstitial space where it can be acquired by neurons (by the same TfR-mediated mechanism) or bound to low-molecular weight ligands (citrate and lactoferrin, among others) for non-transferrin-bound-iron (NTBI) cell uptake (Figure 1). Interestingly, microglia principally take up NTBI facilitated by DMT1 and lactoferrin (Benarroch, 2009; Snyder and Connor, 2009). Typically high in ferritin, microglia are thought to play a role in the maintenance of Fe homeostasis in neurons and when activated, have been shown to release Fe from ferritin mediated by superoxide (Yoshida et al., 1995). To date, the only molecule known to efflux Fe from neurons is Fpn whose internalization in neuronal cells is regulated by Hp and ceruloplasmin (Cp) (Benarroch, 2009; Song et al., 2010; Zheng and Monnot, 2012). Iron in neuronal cells is mostly bound to ferritin or stored in the lysosome. Ferritin is the major Fe-storage protein and is an endogenous iron chelator (Cozzi et al., 2010). An important ferritin sub-type is mitochondrial ferritin (mitFtn), which regulates mitochondrial Fe level and Fe-related oxidization (Arosio et al., 2009). Lysosomes are also important Fe pools, acquiring Fe by turnover of Fe-containing proteins such as cytochrome *c* (Johansson et al., 2010). Due to their acidic nature, lysosomes contain Fe in the more redox-promoting Fe^2+^ state (Terman and Kurz, 2013). Controlling intracellular Fe levels are the iron regulatory protein (IRP)/iron responsive element (IRE) and the hypoxia-inducible factor (HIF)/HIF-responsive elements (HREs) (for review see Wang and Pantopoulos, 2011). Significantly, HREs are present within the TfR1 and DMT1 genes, and HIF regulates Fpn activity by inhibition of Hp (Wang and Pantopoulos, 2011). Recently, Hp was found to cause Fe overload in a rat model of cerebral ischemia (Ding et al., 2011), reflecting Hp’s well-characterized ability to bind to Fpn, inducing its cellular internalization, thereby reducing Fe export (Nemeth et al., 2004).

## Copper and Iron: Peas in a Pod

Like Fe, Cu is a highly useful redox-active metal incorporated into many Cu-containing enzymes such as cytochrome *c* oxidase, SOD-1, monoamine oxidase, and dopamine β-monooxygenase, which play important biological roles (Zheng and Monnot, 2012). Cu is absorbed from the small intestine and delivered to the liver and kidneys where it is predominately (65–90%) bound to Cp (Sharp, 2004). Cellular Cu transport and homeostasis involve the membrane Cu transporters copper transporter-1 (CTR1), DMT1, and Cu exporter ATPases (ATP7A and ATP7B). Delivering Cu to specific intracellular targets are the Cu chaperone proteins antioxidant protein-1 (ATOX1), cytochrome oxidase enzyme complex (COX17), and Cu chaperone for SOD (CCS) (Harris, 2001). Playing a key role in Fe metabolism are the Cu-containing ferrioxidases Cp and hephaestin (Hep). Cp catalyzes the conversion of ferrous iron (Fe^2+^) to ferric (Fe^3+^), which is then transferred to Tf (Sharp, 2004) with Cp, and/or the multi-Cu-centered protein Hep, playing an essential role in the efflux of Fe via Fpn from enterocytes (Sharp, 2004). Significantly, Fpn is highly expressed in the epithelial cells of the choroid plexus and plays a key role in Fe efflux from the CNS (Figure 1). Accordingly, a decrease in Hep activity is suggested to “contribute to iron accumulation in the brain during copper deficiency” (Skjorringe et al., 2012). Additionally, Cu deficiency is known to lower GPI-anchored Cp in mouse/rat spleen and liver – and it is speculated that Cu deficiency may lower GPI-anchored Cp in astrocytes (Mostad and Prohaska, 2011). As noted above, Fe efflux from neurons is mediated chiefly by Fpn whose internalization is regulated by Hp (Nemeth et al., 2004; Song et al., 2010). Conversely, components of the Fe-transport pathway are also Cu responsive. For instance, the Fe transporter DMT1 is also a physiologically relevant Cu transporter (Sharp, 2004). Hence, both Fe and Cu homeostasis can be considered interdependent, with one affecting the other (Figure 1).

## Altered Fe Metabolism in Mutant SOD-1 Cell and Mouse Models

**Figure 2:**
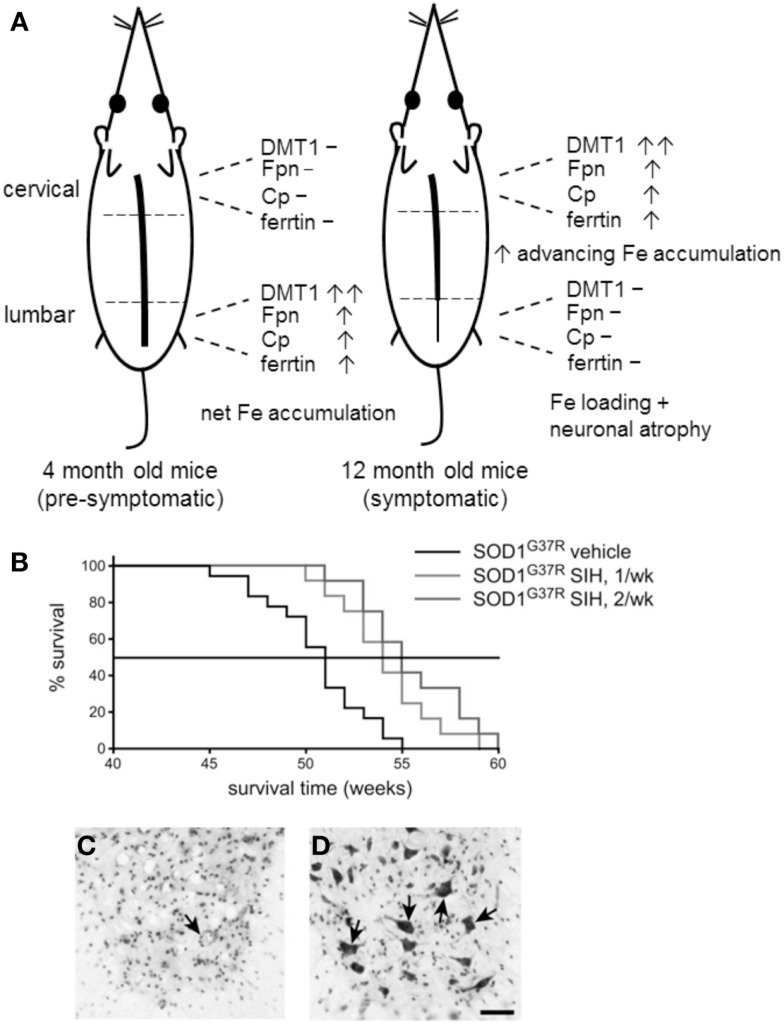
**(A)** The caudal-to-rostral (tail-to-head) pattern of mRNA expression of iron metabolism proteins in young and old SOD-1(G37R) mice provides circumstantial evidence that Fe is involved in the neurodegenerative process. Advanced neurodegeneration and Fe loading is evident in the lumbar region 12-month-old mice, where 8 months earlier, the expression pattern of proteins of Fe metabolism favored net Fe accumulation. **(B)** Treatment of SOD-1(G37R) mice with the Fe chelator SIH extends lifespan in SOD-1(G37R) mice. SOD-1(G37R) mice were given SIH either once or twice a week (*n* = 12) from 8 months of age, a Kaplan–Meier graph shows the percentage of animals surviving with age. **(C,D)** Vehicle control treated 50 week-old SOD-1(G37R) mice showed loss of neurons whereas SIH treatment results in neuronal preservation (arrows) as indicated by cresyl violet-stained lumbar spinal cord tissue sections. Scale bar, 50 μM. [**(B–D)** reproduced with permission and minor modification from Jeong et al., 2009].

A variety of perturbations in Fe metabolism at the mRNA and protein level have been reported in both SOD-1(G37R) and SOD-1(G93A) mutant ALS mice. In late-stage disease (12 months) SOD-1(G37R) mice, a caudal-to-rostral pattern of mRNA expression of a number of proteins involved in Fe uptake and export was seen. Specifically, the mRNA levels of DMT1, TfR1, Fpn, and Cp were highest rostrally (i.e., in the cervical region) compared to the caudal (lumbar) region (Jeong et al., 2009). Western blotting also showed the same expression pattern of these proteins. As DMT1 was expressed at twofold greater levels than either Fpn or Cp, this suggested a potential for greater Fe influx, compared to Fe efflux. Increased caudal-to-rostral spinal cord expression of the Fe-storage protein ferritin was also noted in 12-month-old SOD-1(G93A) mice. Interestingly, the mRNA expression of these proteins in younger pre-symptomatic mice (4-month-old) showed a reverse pattern of DMT1, Fpn, and Cp mRNA expression, i.e., higher lumber but lower cervical expression (Figure 2A), suggesting that Fe accumulation begins before significant neurodegeneration is evident.

These observations correlate to the pattern of Fe deposition and neuronal loss in the spinal cords of these mice, placing Fe “at the scene of the crime,” i.e., at 12 months these mice show advanced neurodegeneration in the lumbar region (where 8 months earlier, Fe began accumulating) but less in the cervical and thoracic spinal cord (where the pattern of dysregulation now favors net Fe accumulation). However, other explanations of neuronal Fe loading are also possible. An *in vivo* model of disrupted axonal transport was also shown to lead to Fe loading in large ventral horn motor neurons (Jeong et al., 2009) and an increase in inflammatory mediators can also result in Fe accumulation (see below). While there is no direct evidence that excess Fe is responsible for neuronal loss, the injurious consequences of excess intracellular Fe are well known. That an Fe chelator was able to limit neuronal loss in this model (see below) also implies that Fe has at least some role in the neurodegenerative mechanism/s. Similar changes in the mRNA expression of these Fe metabolism proteins were also found in SOD-1(G93A) mice, but no Fe loading was detected by Perl’s staining (Jeong et al., 2009).

Considering that disturbances in mitochondrial function are also believed to play a significant role in ALS, it is interesting to note that immunofluorescence staining showed a marked increase in mitochondrial ferritin protein (mitFtn) in the ventral horn motor neurons and astrocytes in 12-month-old SOD-1(G37R) mice but not wild-type mice of the same age mice (Jeong et al., 2009). Overexpression of mitFtn has been shown to trap Fe in the mitochondria, hence Jeong et al. inferred increased mitochondrial Fe and speculated that an increased mitochondrial Fe load in neurons and glia could cause neurodegeneration as occurs in Friedreich’s ataxia (Jeong et al., 2009).

Wang et al. (2011) also examined Fe metabolism in the SOD-1(G93A) ALS model and the ability of Fe chelators to mediate rescue effects (discussed below). Besides finding a 148 and 180% increase in spinal cord Fe in SOD-1 G93A mice (90- and 120-day-old, respectively) compared to age-matched WT mice, these authors also showed induction of TfR1 protein expression. Considering that a major Fe-acquisition pathway in neurons involves intracellular delivery of Fe via the TfR, upregulation of this protein may, in part, explain the increased Fe loading. Additionally, this result also confirms a general dysregulation of Fe homeostasis, as ordinarily, TfR expression decreases in response to increased intracellular Fe (Richardson and Ponka, 1997).

Fe homeostasis in SOD-1(G93A) ALS was further studied using transgenic neuroblastoma SH-SY-5Y cells stably transfected with WT SOD-1 or the G93A mutated human SOD-1 gene (Hadzhieva et al., 2013b). In agreement with studies in SOD-1(G37A) mice, increased mRNA levels of TfR1, ferritin, and DMT1 were found. Additionally, effects on Fe homeostasis within the mitochondrion, a key site for the generation of Fe–sulfur cluster precursor proteins leading to heme biosynthesis, were examined. A 2–3.7 fold increase in mitFtn1&2, iron–sulfur cluster scaffold protein (IScU), and frataxin (Fxn) mRNA was found to also correlate to increased protein expression with the exception of Fxn. Mutant SOD-1(G93A) cells were also significantly Fe loaded compared to WT cells (1.6 fold when normalized to cell number), but interestingly, isolated mitochondria were not Fe-loaded. Further experiments then sought to dissect the mechanism of TfR upregulation. As noted above, Fe deprivation induces classical TfR upregulation via IRE sensing in IRP1 however, Fe supplementation failed to decrease TfR expression, indicating a key dysregulation in Fe homeostasis whereby TfR expression is induced in SOD-1(G93A) mutant cells by IRE/IRP1 independent mechanism/s. ROS is an alternative inducer of TfR expression and has been shown to result in liver Fe loading in alcoholic liver disease (Kohgo et al., 2008). Experiments utilizing H_2_O_2_ and retinoic acid to induce ROS in SOD-1(G93A) mutant cells showed increases in TfR, Ftn, mitFtn1&2, and DMT1 mRNA compared to WT cells, the authors suggesting that ROS-induced upregulation of the Fe importing system may “create a vicious cycle generating strong oxidative stress” (Hadzhieva et al., 2013b).

## Rescue Effects by Fe Chelators in SOD-1 Mouse Models of ALS

Treatment of SOD-1(G37R) ALS mice with the lipophilic Fe chelator SIH increased mean lifespan by 5 weeks (Figure 2B). Histological staining showed that there were more surviving motor neurons in SIH-treated SOD-1(G37R) ALS mice compared with vehicle-treated SOD-1(G37R) mice of the same age (Jeong et al., 2009; Figures 2C,D). In untreated 12-month-old SOD-1(G37R) mice, significant accumulation of Fe was evident in motor neurons of lumbar ventral horn which also appeared atrophied. Glial cells also showed Fe loading. SIH treatment reduced fivefold to sixfold the number of Fe-containing cells detected by Perl’s staining of spinal cord sections, reversed the significant weight loss observed in untreated SOD-1(G37R) mice and improved locomotor function (Jeong et al., 2009).

More recently, Wang et al. (2011) showed that the brain-permeable Fe chelators VK-28 and M30 significantly delayed ALS onset, extended lifespan and reduced spinal cord motor neuron loss in the SOD-1(G93A) transgenic mouse model of ALS. Both Fe chelators significantly attenuated the elevated Fe level and TfR expression, decreased oxygen free radicals and suppressed microglial and astrocytic activation in the spinal cords of SOD-1(G93A) mice (Wang et al., 2011).

Another mechanistic effect of M30 and structurally related chelators is induction of hypoxia-inducible factor-1α (HIF-1α), possibly through enhanced phosphorylation of the p42/44 mitogen-activated protein kinase (MAPK)/ER kinase (MEK) and protein kinase C (PKC) signaling pathways (Avramovich-Tirosh et al., 2010). As a transcriptional activator, HIF-1α is known to regulate a range of neuroprotective signaling pathways and treatment with M30 was shown to increase the gene expression of erythropoietin (EPO), vascular endothelial growth factor (VEGF), enolase 1 and inducible nitric oxide synthase (iNOS) in NSC-34 cells (Kupershmidt et al., 2009), and rat primary embryonic cortical neurons (Avramovich-Tirosh et al., 2010). *In vivo* mice studies with M30 showed upregulation of HIF-1α protein in brain (cortex, hippocampus, and striatum) and spinal cord when given over 30 days. Enhanced HIF-1α levels coincided with increased gene expression of EPO, VEGF, iNOS, glucose transporter 1 (GLUT-1), and heme oxygenase-1 (HO-1) in one or more brain regions but only VEGF showed upregulation in the spinal cord. Additionally, M30 *in vivo* also increased mRNA expression of brain-derived neurotrophic factor (BDNF) and glial cell-derived neurotrophic factor (GDNF) as well as the antioxidant enzymes catalase, SOD-1, and glutathione peroxidase (GPx) in spinal cord and some brain regions (Kupershmidt et al., 2011).

The induction of VEGF by M30 is particularly interesting in light of the many studies demonstrating a neuroprotective role of VEGF in ALS (for review see Llado et al., 2013). Indeed, genetic studies link polymorphisms in the VEGF gene to the development of sALS (Lambrechts et al., 2003; Lysogorskaia et al., 2012) and VEGF therapy has been shown to prevent motor neuron degeneration and increase survival in mutant SOD-1 mice (Storkebaum et al., 2005; Wang et al., 2007). On the basis of these and other observations, VEGF is a suggested therapeutic intervention for ALS. Besides M30, other Fe chelators including desferrioxamine (DFO; the standard clinically used Fe chelator for Fe overload) have also been shown to induce VEGF expression in non-ALS models (Beerepoot et al., 1996).

## Altered Fe Distribution and Metabolism in ALS Patients

In ALS patients, there are several indications of dysregulated iron metabolism. Increased serum ferritin levels and lower Tf (but increased Tf saturation) were recently reported by Nadjar et al. (2012) in a cohort of 694 ALS patients and 297 healthy controls. High serum ferritin was correlated to reduced survival time in ALS patients by 300 days compared to ALS patients with low level serum ferritin. Although serum Fe levels were not significantly different between ALS patients and controls, increased Tf saturation could suggest Fe loading. Previously, Ikeda et al. (2012), assessing a range of serum biomarkers in 92 Japanese ALS patients and 92 age-matched healthy controls, also found that increased serum ferritin correlated with clinical deterioration. Increased ferritin was also reported (Goodall et al., 2008; Qureshi et al., 2008) together with lower serum Tf (Mitchell et al., 2010) in ALS patients. The latter study found that, as biomarkers, higher serum ferritin and lower Tf discriminated between ALS patients and controls with 82% accuracy. These authors also quantified light-chain ferritin (L-ferritin) suggesting that increased L-ferritin might reflect activation, and potentially Fe turnover, in macrophages or brain microglia. Goodall et al. also assessed the levels of C-reactive protein, reporting that high serum ferritin was independent of expression of this inflammation marker.

**Figure 3:**
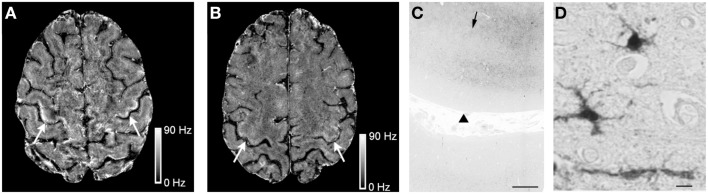
**Iron (Fe) accumulation in the motor cortex of an ALS patient**. The 7T magnetic resonance imaging (MRI) scans reveal Fe accumulation in the motor cortex hand-knob region in a 51-year-old ALS patient **(A)** (arrows), compared to a healthy control. **(B)** Postmortem Fe accumulation in the middle and deeper layers of cortical gray matter and at the gray–white junction by Pearl’s staining (arrowheads indicate the pial surface while arrows indicate the gray–white junction). **(C)** At higher magnification, Pearl’s staining detects Fe in cells with irregular processes suggestive of microglia in the ALS motor cortex. **(D)** Scale bars: C, 1 mm; D, 10 μM. Reproduced with permission and minor modification from Kwan et al. (2012).

High Fe in ALS patients has also been detected by MRI scanning techniques that attribute characteristic T_2_ hypointensities in images of the motor cortex to Fe deposition. Most recently, assessment of 19 ALS cases and 19 healthy controls attributed 3T and 7T FLAIR hypointensities in the hand-knob region of the motor cortex to high Fe which correlated to higher upper motor neuron impairment scores (Kwan et al., 2012; Figures 3A,B). Subsequent postmortem investigations in an ALS patient confirmed that 7T MRI hypointensity changes in the middle and deep layers of the motor cortex correlated to the same regions as a positive Perls’ DAB Fe stain (Figure 3C). Interestingly, higher magnification images seemed to suggest that positive Perl’s DAB staining was present in cells resembling microglia, but was not evident in spinal motor neurons (Kwan et al., 2012; Figure 3D). Immunostaining experiments indicating that excess Fe was stored in ferritin in microglia, as co-localization was shown for ferritin and the microglial marker CD6. It was also noted that luxol fast blue stain showed myelin pallor in the subcortical white matter of the precentral, compared to the postcentra, gyrus (Kwan et al., 2012), which is potentially interesting in light of the recent suggestion by Bartzokis et al. that demyelination may contribute to excess Fe in AD (Raven et al., 2013). Other studies have also attributed changes in T_2_ MRI images to Fe build-up in ALS patients (Oba et al., 1993; Langkammer et al., 2010; Ignjatovic et al., 2013).

Increases in bulk Fe levels had previously been detected in ALS patient ventral horn (Kurlander and Patten, 1979), although not in other studies. Another indication of disturbed Fe distribution may be the presence of higher levels of reactive low-molecular weight Fe species in the CNS. Electron paramagnetic resonance (EPR) spectrometry studies showed that ALS patients had higher levels of inappropriately liganded Fe in the CNS (i.e., bidentate or tridentate ligands that leave spare Fe coordination site/s thereby increasing Fe solubility, in-turn promoting Fe redox cycling and •OH production; Ignjatovic et al., 2012).

## Iron and Neuroinflammation

Microglial activation and astrogliosis are primary hallmarks of the neuroinflammatory environment and widely present in a variety of human neurodegenerative conditions, including ALS (McGeer et al., 1993). They are also a noted feature in mutant SOD-1 mice models of ALS (Jeong et al., 2009; Wang et al., 2011).

Microglia and astrocytes have been shown to play a role in scavenging (and storing) excess Fe, thereby protecting neurons from consequences of Fe loading (Pelizzoni et al., 2013). However, it has been recognized that inflammation modifies the expression of the key Fe-regulating hormone Hp (Nicolas et al., 2002; Ganz, 2003), in turn, down-regulating the Fe export molecule Fpn, and leading to Fe accumulation (Nemeth et al., 2004). This is an element of the classical host Fe-withdrawal defense against infection and inflammation. While there is no direct evidence of a similar process occurring in ALS, interesting clues are provided by studies in dopaminergic neurons. In a study of lipopolysaccharide (LPS)-induced PD in the rat, activated microglia increased expression of the pro-inflammatory cytokines IL-1 and IL-6 and Fe loading was evident in the substantia nigra. Reduced expression of Fpn was also found. In further experiments, SH-SY5Y dopaminergic neurons incubated with media enriched in pro-inflammatory cytokines showed decreased Fpn expression and the appearance of Fe deposits (Zhang et al., 2014). Increased levels of iron regulatory protein 1 (IRP1), transferrin receptor 1 (TfR1), and Hp had previously been observed in IL-1β or tumor necrosis factor alpha (TNF-α) treated ventral mesencephalic neurons (Wang et al., 2013). Additional experiments showed LPS-induced activated microglia led to enhanced IL-1β and TNF-α release upon Fe loading.

Matrix metalloproteinases (MMPs) are also known mediators of neuroinflammation. Increased Fe loading of LPS-stimulated rat microglial (HAPI) cells was shown to enhance secretion of MMP-9 and MMP-1. Increased cellular Fe levels also impaired zymosan phagocytic activity in activated microglia (Mairuae et al., 2011). Others have hypothesized that in addition to activated microglia, Fe maybe deposited in the CNS by infiltrating circulatory monocytes. Once transformed into phagocytic macrophages, and subsequently dying back post-consumption of compromised neurons, these cells may re-release Fe into the labile Fe pool (LIP), enhancing the potential of oxidative stress damage to adjacent cells (including neurons; Andersen et al., 2013).

Collectively, these results are suggestive of the possibility of a vicious cycle whereby Fe loading of microglia and astrocytes increases mediators of neuroinflammation, but in-turn, microglial/astroglial Fe loading results from inflammation-mediated suppression of Fpn by Hp. As yet, there is no direct evidence of these processes occurring in ALS, but processes similar to these may provide a potential explanation for the characteristic T_2_ hypointensities in MRI images of the motor cortex due to Fe deposition and increased serum ferritin in ALS patients. These observations also underscore the general anti-inflammatory property of Fe and Cu chelators in murine SOD-1 ALS models, as evidenced by reduced microglial activation (Jeong et al., 2009; Kupershmidt et al., 2011).

## Copper in ALS

Most interest in Cu in ALS centers on mutations in SOD-1 and over 160 mutations have been associated with ALS (Sreedharan and Brown, 2013). Formation of toxic SOD-1 aggregates is strongly implicated (Chattopadhyay and Valentine, 2009; Sreedharan and Brown, 2013) and multiple data describe defective Cu (and Zn) binding by various SOD-1 mutants (Carri et al., 1994; Eum and Kang, 1999; Hayward et al., 2002). Early studies showed that the Cu chelators diethyldithiocarbamate and penicillamine could reverse mutant SOD reactivity (Wiedau-Pazos et al., 1996). Undermetallation of SOD-1 seems to be a significant factor in SOD-1 aggregation *in vivo* (Hayward et al., 2002; Lelie et al., 2011). Studies in SOD-1 mutant mice showed that SOD-1 fractions isolated from spinal cords mutant SOD-1 mice that were insoluble (i.e., aggregated) were Cu and Zn depleted, whereas soluble SOD-1 fractions were highly metalated (Lelie et al., 2011). A re-distribution of Cu from gray to white matters correlated to areas of high SOD-1 and increased Zn was also found in the white matter in mutant SOD-1 mice (Lelie et al., 2011). In a study of mutant H43R SOD-1, a rapidly progressing form of fALS, metal-replete (halo) H43R SOD-1 exhibited a stable β-barrel structure, but apo-H43R SOD-1 was unstable and readily misfolded (Fujimaki et al., 2013). Spectroscopic studies showed that Cu^2+^ binding differed between the halo- and apo-forms, the authors suggesting that bound Cu^2+^ in the apo-H43R form was pro-oxidant. While SOD-1 metalation status appears important in promoting aggregation, failure of SOD-1 maturation, due to disrupted formation of a stabilizing intra-subunit disulfide bond also, appears to be significant (Seetharaman et al., 2009). Irrespective of the precise aggregation mechanism, SOD-1 aggregates are thought generally to acquire a “toxic gain of function” related to promotion of oxidative stress. Impairment of axonal transport (De Vos et al., 2007) and mitochondrial dysfunction (Li et al., 2010) could also be sequelae. More recently, SOD-1 mutants were found to interfere with protein transport between the ER–Golgi apparatus, leading to Golgi fragmentation and induction of ER stress (Atkin et al., 2013). Others have suggested that palmitoylation (a reversible post-translational modification that influences structure, function, and localization) of SOD-1 mutants could increase their targeting to cellular membranes, potentially increasing their propensity to cause mitochondrial dysfunction or ER stress (Antinone et al., 2013).

Other Cu-containing enzymes or chaperones showing modulations of expression or activity in ALS patients include Cp and MT. An early Polish study assessing the levels of Cp in 14 ALS patients found suppressed levels in eight patients (Domzal and Radzikowska, 1983) but this was not replicated in other studies (Boll et al., 2008; Goodall et al., 2008). However, a recent study of Cp isoforms in ALS showed that patients had higher abundance of non-sialylated protein forms of Cp, indicating “a Cp functional impairment” (Conti et al., 2008). Further evidence suggestive of role for Cp emerged from a proteomic 2D difference-in-gel electrophoresis analysis of CFS in ALS patients (Brettschneider et al., 2010). Compared to controls, only six proteins were found to be modulated between these groups, the ceruloplasmin precursor protein (CpPP) and the Fe delivery protein Tf were two proteins that were downregulated (Brettschneider et al., 2010).

Studies with the endogenous antioxidant chaperone MT also provide insight into the important role played by Cu in mutant SOD-1 ALS. In a cohort of 12 ALS patients, the immunohistochemical expression of the MT-1/2 isoform was significantly reduced in the spinal cord relative to the control group, although both MT-1/2 and MT-3 were found in glia (Hozumi et al., 2008). Expression of MT-3 in astrocytes in the gray matter of the lumbar spinal cord negatively correlated with ALS duration. Additionally, patients with MT-3-positive neurons also showed MT-3 positive glia. Previous studies had shown that SOD-1(G93A) mice with either MT-1/2 or MT-3 knocked-down had accelerated ALS disease course (Nagano et al., 2001) and recently SOD-1G93A mice engineered to also overexpress MT showed significantly enhanced survival together with reduced motor neuron loss and degeneration of ventral root axons and skeletal muscle atrophy. Glial activation was also reduced. Additionally, SOD-1 aggregates in the spinal cord glia were reduced in double transgenic mice (Tokuda et al., 2014).

Aside from its role in SOD-1 and other metalloenzymes or chaperones, there is some evidence of general Cu accumulation in human ALS. Most recently, 52 ALS patients showed a significant (*p* < 0.05) increase in Cu in CSF (Roos et al., 2013), but no excess of Cu was found in CSF by others (Boll et al., 2008). Slightly elevated Cu and Zn were also found in erythrocytes in SOD-1 fALS patients (Vinceti et al., 2002). In a study of spinal cord tissue from sALS patients found a significant Cu increase (Kurlander and Patten, 1979). Accumulations of Cu have also been noted in the SOD-1(G93A) mouse (Li et al., 2006; Tokuda et al., 2013), prompting assessment of various Cu chelators in this model, including d-penicilamine (Hottinger et al., 1997) and trientine (Nagano et al., 2003). Most recently, the Cu chelator tetrathiomolybdate (TM) was shown to significantly improve symptoms and survival in SOD-1(G93A) ALS mice (Tokuda et al., 2008) by removal of Cu from the Cys_111_ residue in mutant SOD-1 but not from histidyl sites essential for SOD-1 enzymatic activity, thereby preventing “aberrant copper chemistry.” Subsequently, an almost threefold accumulation of Cu in spinal cords of SOD-1(G93A) mice was reversed by TM treatment and modulations in expression of a variety of Cu-containing proteins in SOD-1 mutants were reported (Tokuda et al., 2013). Specifically, in the G93A, G127X, G85R, and D90A SOD-1 mutants, western blots showed significantly increased expression of Steap2, Atox1, CTR1, and CCS (all except the G127X mutant) and significant decrease in Atp7a in spinal cord tissue. The functional significance of these modulations was described as skewing the Cu-transport system toward Cu accumulation in the spinal cord (Tokuda et al., 2013).

Additional support for a deleterious role of Cu in mutant SOD-1 ALS was provided by studies with Tg mice engineered to contain both SOD-1G86R and mottled/brindled (MoBr) mutations (Kiaei et al., 2004). The latter is a model of Menkes disease, a human genetic disorder causing copper deficiency. In SOD-1G86R/MoBr mice spinal cord, Cu was reduced by 60%, and these mice lived 9% longer than mice bearing the SOD-1G86R mutation alone, supporting a role for Cu in SOD-1-related fALS.

## Endoplasmic Reticulum Stress: An Under-Recognized Role for Iron?

Triggers of ER stress include Ca^2+^ storage defects, lipid/glycolipid imbalances, oxidative stress, and a variety of environmental insults such as viral infections and drug exposure (Rutkowski and Kaufman, 2007). In ALS, major ER stress inducers are misfolded SOD-1, TDP-43, and/or FUS proteins that accumulate in the ER thereby inducing the unfolded protein response (UPR) (Atkin et al., 2008).

**Figure 4:**
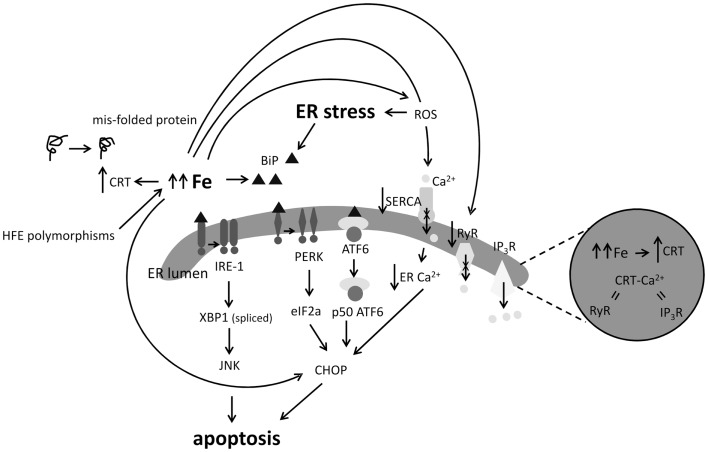
**Impact of excess iron (Fe) on indicators of ER stress and calcium (Ca^2+^) signaling**. ER stress results in the activation of the IRE1, PERK, and ATF6 apoptotic pathways. Loss of BiP induces oligomerization of IRE1 or PERK or activates ATF6 which downstream results in JNK or CHOP mediated apoptosis. High Fe in cells and animal models has been shown to induce BiP, CHOP, and calrectulin (CRT) expression. Excess Fe and resultant redox processes may inhibit function of the SERCA Ca^2+^ pump, decreasing ER Ca^2+^ levels, and leading to CHOP induction, on the other hand, Fe has been shown to inhibit the ryanodine receptor (RyR) ER Ca^2+^ exporter. Induction of CRT by Fe could suggest that Fe participates in causing protein mis-folds or results in perturbed Ca^2+^ balance in the ER.

The UPR acts to reduce ER stress by re-establishing normal protein folding and proceeds by the activation of three distinct ER stress transduction pathways following removal of the ER chaperon binding immunoglobulin protein (BiP) from the luminal domains of inositol-requiring enzyme-1 (IRE1), protein kinase R (PKR)-like endoplasmic reticulum kinase (PERK), and activating transcription factor 6 (ATF6). However, severe or chronic UPR triggers apoptosis (Bertolotti et al., 2000; Figure 4).

In human ALS patients, a variety of indicators of ER stress leading to induction of the UPR have been identified. A group of 12 sALS patients showed a 26-fold higher positive immunostaining for the ER chaperone protein GRP78 (also known as BiP) compared to controls (Sasaki, 2010) and in the spinal cords of sALS patients, UPR induction was demonstrated by upregulation of stress sensor kinases (IRE1, ATF6, and PERK), chaperones protein disulfide isomerase (PDI), ER protein 57 (Erp57), apoptotic mediators CCAAT/-enhancer-binding protein homologous protein (CHOP), and caspase 4 (Atkin et al., 2008).

It is well appreciated that Fe overload increases intracellular ROS (Galaris and Pantopoulos, 2008), a known initiator of ER stress. More specific observations of the link between Fe and ER stress include upregulation of the ER chaperons BiP and calreticulin (CRT) in Fe-loaded astrocytoma (Ye and Connor, 2000); increased BiP expression in the livers of dietary Fe-loaded mice (Petrak et al., 2007); reduced mRNA and protein expression of the major blood Fe transporter Tf was found in transfected Hep 3B hepatoma cells overexpressing CHOP (You et al., 2003; Figure 4). Mutations in the hemochromatosis gene (HFE) have also been associated with sALS. Both the H63D and C282Y HFE polymorphisms interfere with the ability of HFE to limit Fe uptake from Tf, which results in Fe loading (Feder et al., 1998; Lee et al., 2007). Recently, human neuronal SH-SY5Y cells with a tetracycline-controlled FLAG-tagged HFE H63D mutation showed upregulation of the UPR markers BiP, PDI, and IRE1α after tetracycline treatment (Liu et al., 2011b). Induction of the master Fe-regulating hormone Hp by ER stress has also been noted (Vecchi et al., 2009) with subsequent downstream effects on the expression of the cellular Fe exporter, Fpn, and ferritin. These studies were the first to demonstrate the reciprocal relationship between Fe homeostasis and ER stress, describing Hp induction as a marker of ER stress (Vecchi et al., 2009).

## Intracellular Calcium Signaling is also Responsive to Fe

Calcium (Ca^2+^) storage defects are thought to be significant in ALS and another potential consequence of excess Fe in ALS may be detrimental modulation of Ca^2+^ signaling. Early observations showed that the ROS generating Fe^2+^/H_2_O_2_ couple decreased Ca^2+^ uptake by skeletal sarcoplasmic reticulum (SR) (Stoyanovsky et al., 1994; Castilho et al., 1996) by inhibiting the activity of the Ca^2+^ pump Ca^2+^-ATPase (SERCA) (Moreau et al., 1998) which transfers Ca^2+^ from cytosol into SR lumen (Figure 4). However, Fe^2+^ given alone enhanced Ca^2+^ uptake into rat heart SR vesicles, mimicking ruthenium red by direct inhibition of the ryanodine receptor (RyR), one of the channels by which Ca^2+^ is released from the SR (Kim et al., 1995). Interestingly, the ER stress inducer thapsigargin also inhibits SR Ca^2+^-ATPase (thereby reducing ER lumen Ca^2+^ uptake) and was shown to increase Fe uptake in K562 leukemic cells with depletion of intracellular Ca^2+^ or chelation of extracellular Ca^2+^ resulting in inhibition of Fe uptake (Ci et al., 2003).

Additionally, Fe was shown to be necessary for Ca^2+^ signal generation and ERK1/2 stimulation induced by the glutamate agonist *N*-methyl-d-aspartate (NMDA), as Fe chelation reduced Ca^2+^ signal duration and prevented NMDA-induced ERK1/2 activation (Munoz et al., 2011). Fe supplementation of primary hippocampal neurons kept in Ca^2+^-free medium also elicited Ca^2+^ signals, suggesting that hippocampal neurons require Fe to generate RyR-mediated Ca^2+^ signals after NMDA receptor stimulation (Munoz et al., 2011). Hp treatment of human osteoblasts was also shown to increase intracellular Ca^2+^, an effect potentiated by added Fe (Li et al., 2012). Pretreatment of osteoblasts with a L-type Ca^2+^ channel blocker or a RyR antagonist inhibited Ca^2+^ release from the SR, demonstrating that the increase of intracellular Ca^2+^ induced by Hp probably reflected Ca^2+^ release from the ER, triggered by Ca^2+^ influx (Li et al., 2012). Interestingly, mRNA and protein expression of the ER chaperone CRT that plays a key role in protein folding protein in the ER, was shown to increase with Fe concentration (Liu et al., 2011a), suggesting that CRT induction may be part of the cellular response to excess Fe, which in turn could affect ER Ca^2+^ buffering. Considering that CRT functions as a misfolded protein chaperone, CRT induction could suggest that excess Fe may also negatively impact protein folding.

## A Role for Metals in Toxic TDP-43 Accumulations?

Neuronal cytoplasmic inclusions of TDP-43 are a major component of ubiquitinated protein aggregates found in the CNS of sporadic MND patients. Indeed, TDP-43 inclusions are a histopathological feature in the vast majority of MND patients (Da Cruz and Cleveland, 2011). Current thinking suggests these inclusions mediate neuronal damage by inducing ER stress or by affecting RNA processing (Atkin et al., 2008).

Interestingly, several lines of evidence also point to the role for metals in TDP-43 accumulation and toxicity. In SH-SY5Y neuronal-like cells expressing endogenous TDP-43, Zn treatment reduced TDP-43 expression but enhanced the formation of TDP-43 positive inclusions (Caragounis et al., 2010). However, addition of Cu or Fe did not induce similar effects, suggesting that specific Zn-associated processes might modulate TDP-43 accumulation. Additionally, treatment of SOD-1(G93A) mice with the Cu-containing chelate, Cu-ASTM, prevented the accumulation of abnormally phosphorylated and fragmented TDP-43 in the spinal cord (Soon et al., 2011). The Fe chelator M30 and a structural analog also showed reduced TDP-43 accumulation in the SOD-1(G93A) model (Wang et al., 2011). Another clue emerged from a phenotypic screen for small molecules that could reverse TDP-43 toxicity in yeast. One class of “hit” compounds were 8-hydroxyquinolines, biologically active metal chelators structurally related to clioquinol, the authors suggesting that 8-hydroxyquinolines may be able to target TDP-43 accumulation by altering metal homeostasis (Tardiff et al., 2012). It was also recently reported that mice bearing the TDP-43(A315T) mutation had increased Cu, Zn, and manganese (Mn) in the spinal cords not evident in wild-type mice (Dang et al., 2014). Increased levels of the monocyte chemoattractant protein-1 (MCP-1) in TDP-43(A315T) mouse spinal cord indicated microglial activation and inflammation. Protein oxidation was also higher in TDP-43(A315T) spinal cord and is a hallmark of metal-mediated Fenton chemistry. How and why TDP-43 inclusions alter the metallic landscape of the spinal cord in SOD-1 ALS will be an on-going research interest.

## Discussion

The transition elements Fe and Cu are involved in ALS progression by potentially multiple mechanisms. A clear role is evident for Cu and Zn in the key enzyme SOD-1. Mutations affecting the metal-binding sites may lead to structural instability, possibly through loss of integrity of a intra-subunit disulfide bond, and then leading to accumulation and/or to a toxic gain of function. Other Cu-containing enzymes may also be relevant in ALS progression. Early data implicated reduced Cp expression, but this was not reproduced in other studies, but higher levels of non-sialylated Cp were detected in another study in ALS patients and could indicate a Cp functional impairment. Reduced expression of MT has also been noted in ALS patients and in SOD-1(G93A) models. Conversely, MT overexpression or treatment rescues phenotype in mutant SOD-1 mice. Studies in patients and SOD-1 mice also show bulk increases in Cu in the spinal cord. That a variety of Cu chelators also extend lifespan and reverse pathophysiological effects in SOD-1 mouse models, provide evidence suggestive a role for Cu in ALS progression.

In sALS patients, high serum ferritin levels have been consistently shown to correlate with ALS progression and characteristic MRI T_2_ shortening shows Fe overload in the motor cortex of ALS patients. To what extent this excess Fe drives the ALS disease process is unknown (although there are some clues provided by SOD-1 models) and it is likely that excess Fe loading represents a process secondary to primary disease mechanism/s.

As a biomarker, increased serum ferritin may infer increased body iron stores, but not necessarily so, as infection or inflammation can also induce its expression. However, increased Fe and inflammation are linked and one exacerbates the other creating a vicious cycle. Indeed, a relatively good case, albeit circumstantial, can be made that excess Fe in ALS is a consequence of inflammatory processes. That Goodall et al. reported increased serum ferritin in ALS patients in the absence of an increase of the inflammatory marker C-reactive protein, does not necessarily rule out a role for inflammation in serum ferritin induction (and Fe deposition), as more discrete localized inflammation could have occurred that did not generate a detectable serum trace. However, increases in Tf saturation, in addition to high serum ferritin reported by Nadjar et al., also point to excess Fe. Besides inflammation, another potential mechanism that could cause Fe loading in ALS could be reduced Cp expression or activity, as Cp plays a role in mediating Fe export by Fpn.

The form in which Fe is present also influences its degree of danger. Low-molecular weight labile Fe is more hazardous than Fe stored in ferritin. But Fe in ferritin is still bioavailable and can readily be released by activated microglia in a process mediated by superoxide. Increased labile Fe, or “inappropriately chelated” Fe, reported by Ignjatovic et al. in ALS patient CSF also suggests that excess Fe in ALS is present in a form that can readily contribute to redox stress.

As models of fALS, mutant SOD-1 mice reproducibly show Fe loading in spinal cord, and in SOD-1(G37R) mice, dysregulation of Fe metabolism resulting in neuronal Fe loading precedes significant neuronal loss, providing at least circumstantial evidence that Fe is involved in the neurodegenerative process. The ability of a variety of structurally diverse Fe chelators to extend lifespan, prevent neuronal loss and mediate other rescue effects provides additional evidence that Fe is probably involved in the neurodegenerative process in ALS.

Induction of ER stress due to aggregation of protein mis-folds in ALS is gaining traction as a major mechanism of neuronal loss. Various studies describe how excess Fe may increase ER stress, as Fe loading causes increases in the expression of a variety of ER chaperones. Perturbed intracellular Ca^2+^ signaling can also be an expected consequence of Fe loading due to inhibition of ER Ca^2+^ pumps. Another link to ER stress and transition metals is provided by recent studies showing enrichment of TDP-43 aggregates with Cu, Zn, and other metals. In the context of a system already under stress, it is intriguing to speculate that transition metal loading in ALS may also be enhancing ER stress. However, no direct evidence yet exists for this hypothesis.

While the collective evidence is strengthening, the insult that excess transition metals render in ALS still requires clarification. However, as a facet of a complex disease, lowering (or modulating) levels of transition metals, particularly Cu and Fe, in ALS would seem to be an avenue worth pursuing.

## Author Contributions

David B. Lovejoy and Gilles J. Guillemin researched and wrote the paper.

## Conflict of Interest Statement

The Associate Editor Dr. Chung declares that, despite having collaborated with author Dr. Guillemin, the review process was handled objectively and no conflict of interest exists. The authors declare that the research was conducted in the absence of any commercial or financial relationships that could be construed as a potential conflict of interest.
